# HSP70 overexpression may play a protective role in the mouse embryos stimulated by CUMS

**DOI:** 10.1186/s12958-015-0123-z

**Published:** 2015-11-16

**Authors:** Xiao-Hong Li, Hou-Qing Pang, Lang Qin, Song Jin, Xun Zeng, Yu Bai, Shang-Wei Li

**Affiliations:** Reproductive Medicine Center, West China Second Hospital of Sichuan University, Chengdu, Sichuan China; Department of Ultrasonography, West China Second Hospital of Sichuan University, Chengdu, Sichuan China

**Keywords:** Heat shock protein70, Chronic unpredictable mild stress, Embryos, Immunofluorescence, Real-time polymerase chain reaction

## Abstract

**Background:**

We evaluated whether heat shock protein HSP70 plays a protective role in the embryos of Kunming mice subjected to chronic unpredictable mild stress.

**Methods:**

Female mice were stimulated for 4 weeks with nine stressors and then divided into mild, moderate and severe stress groups. Superovulation was induced with a gonadotropin preparation (PMSG/HCG) and HSP70 expression in 2-cell embryos and day 4 embryos was detected by immunofluorescence (IF) and real-time polymerase chain reaction (RT-PCR).

**Results:**

In the mild stress group, ovarian response and oocyte development potential were similar to those of the control group, while the HSP70 mRNA levels of the embryos were significantly higher (*P* < 0.05). In the severe stress group, ovarian response and oocyte development potential decreased compared with the control group (*P* < 0.05), while the HSP70 mRNA levels were similar. The results of the moderate stress group were intermediate among the three groups. Furthermore, HSP70 mRNA levels of the embryos were shown to be positively associated with parameters of oocyte and embryo development potential (*P* < 0.05).

**Conclusions:**

HSP70 overexpression may play a protective role in the embryos of the mild or moderate stress mice stimulated by chronic unpredictable mild stress.

## Background

Stress is a complex state of threatened homeostasis, which mobilizes a composite spectrum of nervous, endocrine, and immune system responses to restore and maintain homeostasis. In the neuroendocrine network, the reproductive endocrine system is not only involved in the response to stress, but is more vulnerable to stress damage than other systems. Studies have confirmed that stress can affect the human menstrual cycle, leading to primary dysmenorrhea, premenstrual syndrome and hypothalamic amenorrhea [1–3]. Chronic psychosocial stressors are detrimental to the ovarian reserve of infertile women [4–7], and stressful life events are associated with poor in vitro fertilization outcomes [8–10]. Both infertility and its treatment using assisted reproductive technology (ART) are stressful. Couples often describe the experience of infertility as a critical, significant life event associated with emotional challenges [11–13]. Some infertile patients undergoing ART have reported high levels of depressive symptoms, anxiety and distress [14–18]. A meta-analysis found that stress, trait anxiety and state anxiety had negative associations with clinical pregnancy rates for patients undergoing ART [8].

Although animal models cannot replicate human psychopathology in every detail, they offer the possibility of evaluating the main effects and interactions in a controlled, prospective manner. Chronic unpredictable mild stress (CUMS) is one of the behavioral models used to simulate human depression in some respects, such as loss of normal aggressiveness [19]. This animal model is consistent with ethical principles for scientific experiments on animals and has validity [20]. Our research group established the CUMS animal model with Kunming mice for the first time. We found that after the administration of pregnant mares’ serum gonadotropin (PMSG) and human chorionic gonadotropin (hCG) to induce superovulation, the numbers of oocytes and embryos obtained from the mice decreased under CUMS conditions. Wu et al. [21, 22] also reported that CUMS inhibited follicular development and increased follicular atresia.

There are clinical cases reporting successful pregnancies in women in a stressed state [18], so we inferred that their reproductive biology might be protected against stress factors. Heat shock proteins (HSPs) are highly conserved cellular stress proteins present in every organism from bacteria to humans. They were first described in 1962 as leading to a puffing pattern on *Drosophila* polytene chromosomes after thermal stress [23]. As a member of the HSP superfamily, HSP70 may act as a molecular chaperone, and assist in the cell stress response by resisting apoptosis and oxidative stress, and regulating immune responses [24]. Apart from expression in tumors and sensitive organs such as the brain and heart, HSP70 is also expressed in the ovaries, endometrium, decidua, placenta and amniotic fluid, and it may play a role in oogenesis and embryogenesis. The body may protect its reproductive function by increasing the expression of HSP70 when subjected to thermal and oxidative stressors [25]. However, no studies have yet investigated the levels of HSP70 in the female reproductive system under psychological stress.

This study aimed to explore the expression of HSP70 in the response of embryos in a Kunming mouse model of CUMS following ovarian superovulation.

## Methods

### Animals

This study was carried out in strict accordance with the recommendations in the Guide for the Care and Use of Laboratory Animals of the National Institutes of Health. The protocol was approved by the Committee on the Ethics of Animal Experiments of Sichuan University (Permit Number: AE2014034). All surgery was performed under sodium pentobarbital anesthesia, and all efforts were made to minimize suffering. Female Kunming mice (4–5 weeks old) were obtained from the Animal Service Center of Sichuan University and were assigned by random number table to two groups: an unhandled control group and the experimental CUMS group. Mice were housed five per cage and were acclimatized to the animal colony for one week under the following conditions: standard rodent diet and tap water *ad libitum*, a 12/12 h light/dark cycle (lights on 07:30–19:30 h), and a constant temperature of 21–22 °C and humidity of 55–65 %. The experimental group then underwent a 4-week CUMS procedure, while the control group continued with the same conditions used during acclimatization.

### Stress induction

There were 150 mice in the experimental group and 50 in the control group. The CUMS protocol was adapted from our former method and revised [26]. The experimental mice were stimulated for 4 weeks with one of the nine stressors in the same order each day. The nine stressors were as follows: (1) 24-h damp bedding and cage tilting (cages were tilted to 45° from the horizontal); (2) 4-h restraint; (3) 5-min swimming in an ice bath; (4) 24-h food deprivation; (5) 24-h water deprivation with empty drinking bottles; (6) 24-h social isolation (one mouse per cage); (7) 5-min heat stress in an oven at 40 °C; (8) 1-min tail clamping; (9) 24-h exposure to strange objects such as plastic cups, spoons, or pieces of cloth. Each stressor was carried out in each experimental mouse 3 or 4 times in the 4-week CUMS.

### Open field test (OFT) and sucrose consumption

After 4 weeks of CUMS, we applied an OFT and a sucrose consumption test to the experimental and control mice. For the OFT, we constructed a box measuring 60 × 60 × 40 cm with matte white acrylic, placed a mouse inside and measured the time spent in the center, any distance moved (number of cross lattices), rearing (vertical) activity, grooming time, and defecation (number of fecal boli) over 5 min [27]. In the sucrose consumption test, we calculated each mouse’s consumption of 2 % sucrose in 4 h. We also measured the body weight of the experimental and control mice before and after the 4-week CUMS.

### Division of experimental group

According to the data of the OFT and the sucrose consumption test, we found that the time spent in center, the distance moved, rearing count and defecation of the OFT and the sucrose consumption were different between the experimental group and the control group. Apart from the narrow range of the defecation and sucrose consumption, we chosen three indicators of the OFT including the time spent in center, distance moved, rearing count to divide stress levels of experimental mice. The cut off values of the time spent in center in mild, moderate and severe stress groups were 8 s and 11 s, respectively. The cut off values of distance moved in mild, moderate and severe stress groups were 75 and 90 cross lattices, respectively. The cut off values of rearing count in mild, moderate and severe stress groups were 14 and 11 times, respectively.

So, we defined the mild stress mice as: time spent in the center was 6–8 s, or the distance moved was 90–105 cross lattices, or the rearing count was 14–16 times. Moderate stress mice were defined as: time spent in the center was 9–11 s, or the distance moved was 75–89 cross lattices, or the rearing count was 11–13 times. Severe stress mice were defined as: time spent in the center was 12–14 s, or the distance moved was 60–74 cross lattices, or the rearing count was 8–10 times. These mice were also defined as severe stress ones if they had at least one indicator that is in line with the severe standards, and so on.

### Collection of mouse embryos

The mice were injected intraperitoneally with 10 IU PMSG (Animal Center of Tianjin, P. R. China) at 4 pm, followed by an injection of 10 IU of hCG (Biochemical Pharmaceutical Factory of Shanghai, P. R. China) 46–48 h later. After the second injection, females were placed two per cage with fertile male mice. The day of sighting a vaginal plug was designated as day 1 (D1) *post coitum* (pc). Mated females were separated from males on D1 pc. Their 2-cell embryos were obtained at 2 pm D2, and their morula-early blastocysts were obtained on D4.

High-quality embryos on D4 were defined as those classifiable as grade I–III according to the published criteria for human embryos [28].

### Real time polymerase chain reaction (RT-PCR)

RNA was extracted from 50 2-cell embryos or 30 D4 embryos collected from the mice *in vivo*, using RNeasy Micro Kits (Qiagen Inc., Germany) according to the manufacturer’s instructions. β-actin was a housekeeping gene for normalization of this data. The RT-PCR reactions were performed in a final volume of 20 μL, containing 10 μL of SYBR Green master mix (Roche Diagnostics, Indianapolis, IN, USA), 3 μL of H_2_O, 1 μL each of forward primer (GAGGAGTTCAAGAGGAAG) and reverse primer (TGATGGATGTGTAGAAGTC) and 3 μL of cDNA template or water (non-template negative control). An ABI 7500 thermal cycler (Applied Biosystems, Foster City, CA, USA) was employed for RT-PCR amplification, which was performed under the following conditions: one cycle of 95 °C for 10 min; 45 cycles of 95 °C for 15 s, 56 °C for 30 s, 72 °C for 30 s; and a final cycle of 95 °C for 15 s, 60 °C for 15 s, and then a gradual increase to 95 °C over 30 min at a ramp rate of 2 % for melting curve analysis.

### Immunofluorescence (IF) staining

For IF localization of HSP70, 2-cell and D4 embryos were fixed in freshly prepared 4 % paraformaldehyde (Sigma-Aldrich, St Louis, MO, USA) in phosphate-buffered saline (PBS) for 1 h at room temperature (RT), washed in PBS supplemented with 2 % (w/v) bovine serum albumin (Sigma-Aldrich; PBS-BSA) by pipetting through three sequential 50 μL drops, which were then transferred to 0.1 M glycine (Sigma-Aldrich) in PBS-BSA for 5 min at RT to neutralize aldehydes. After washing again in PBS-BSA (as above) embryos were permeabilized in 0.1 % Triton X-100 in PBS for 10 min at RT, then washed a third time in PBS-BSA. The embryos were incubated overnight at 4 °C with a 1:100 dilution of mouse monoclonal anti-HSP70 antibody (ab5439; Abcam). Negative controls were treated with PBS-BSA alone. Embryos were then washed and incubated with a 1:100 dilution of goat anti-mouse IgG (Beijing Zhongshan Jinqiao Biotechnology Co., Ltd., Beijing, P. R. China). Embryos were washed and counterstained with Hoechst 33258 (0.5 μg/mL; Santa Cruz Biochemical Co., Santa Cruz CA, USA) to stain the cell nuclei.

### Statistical analyses

Measurements are shown as the mean ± standard deviation (SD) and analyzed by Student’s *t* test, ANOVA and the LSD test. Numeric data such as the rates of fertilization and development of high quality embryos were evaluated with the *χ*^2^ test and the partitioning *χ*^2^ method. Correlations were analyzed using linear correlation. All statistical analyses were performed using SPSS version 17.0 (IBM Corp., Armonk, NY, USA) and significance was defined as *P* < 0.05.

## Results

### Validation of the mouse CUMS model by OFT and sucrose consumption test

After 4 weeks of CUMS, the body weight of the experimental mice was 29.5 ± 4.7 g, which was significantly lower than the control group (33.9 ± 5.1)(*P* < 0.05).

Data obtained on the time spent in the center of the box, distance moved, rearing, grooming and defecation activities in an open field are shown in Table 1. Compared with the control group, the time spent in the center and amount of defecation in stressed mice increased significantly (*P* < 0.05), while the distance moved and amount of rearing decreased significantly in the stressed mice (*P* < 0.05). After 4 weeks of CUMS, the sucrose consumption of the stressed mice was significantly less than in the control group (*P* < 0.05; Table 1).

**Table 1:** Open field test and sucrose consumption test results after 4 weeks of chronic unpredictable mild stress (CUMS)

	Control group	Stressed group	*P* value
No. of mice	50	150	
Body weight (g)	33.9 ± 5.1	29.5 ± 4.7	<0.05
Time spent in center (s)	4 ± 1	10 ± 4	<0.05
Distance moved (no. of cross lattices)	166 ± 38	81 ± 21	<0.05
Rearing count	39 ± 9	12 ± 4	<0.05
Grooming count	3 ± 1	4 ± 1	NS
Defecation (no. of fecal boli)	2 ± 1	4 ± 2	<0.05
Sucrose consumption (g)	4 ± 1	2 ± 1	<0.05

Data are shown as the mean ± SD and were analyzed using Student’s *t* tests. *NS* not significant

According to the results of the time spent in the center, distance moved and rearing activities, we divided the experimental mice into mild, moderate and severe stress groups. Among the 150 experimental mice, there were 75 mild stress mice (50 %), 55 moderate stress mice (37 %), and 20 severe stress mice (13 %).

### CUMS decreased the ovarian response to superovulation

Table 2 shows the influence of CUMS on the development potential of oocytes and embryos after ovarian hyperstimulation. The numbers of retrieved oocytes, 2-cell embryos, D4 embryos including high quality embryos and blastocysts, fertilization rate, and high quality embryo rate at D4 among the moderate and severe stress groups compared with the control group were significantly different (*P* < 0.05, Table 2), while the mild group was not significantly different to the control group. Further statistical analysis showed that compared with the control group, these variables were all significantly lower in the moderate or severe stress groups.

**Table 2:** Development potential of oocytes and embryos after 4 weeks of CUMS

	Control group	Mild stress group	Moderate stress group	Severe stress group	*P* value
No. of mice	20	20	20	20	
No. of oocytes^a^	32 ± 7	29 ± 5	22 ± 6*	15 ± 5*	<0.05
Fertilization rate^b^	87 %	78 %	61 %*	40 %*	<0.05
No. of 2-cell embryos^a^	27 ± 5	22 ± 4	15 ± 5*	9 ± 2*	<0.05
No. of D4 embryos^a^	24 ± 4	18 ± 3	10 ± 4*	4 ± 2*	<0.05
No. of high quality embryos^a^	20 ± 5	13 ± 4	5 ± 2*	1 ± 1*	<0.05
High quality embryo rate^b^	83 %	72 %	50 %*	25 %*	<0.05
No. of blastocysts^a^	8 ± 3	5 ± 2	2 ± 1*	1 ± 1*	<0.05
Blastocyst information rate^b^	33 %	28 %	20 %	25 %	NS

^a^Data are shown as the mean ± SD and were analyzed by ANOVA and LSD testing

^b^Data are shown as rates and were analyzed by *χ* ^2^ test and the partitioning *χ* ^2^ method

*P < 0.05 compared with the control group, measured by LSD test or the partitioning *χ* ^2^ method. *NS* not significant

### Expression of HSP70 in 2-cell and D4 embryos after CUMS

**Fig. 1:**
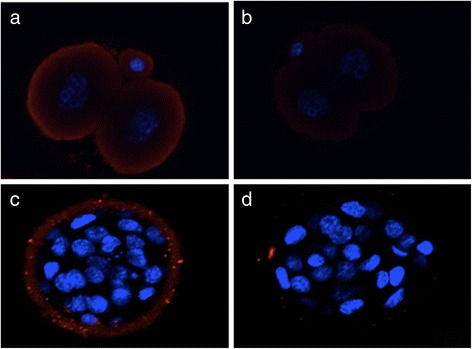
Expression of HSP70 in 2-cell embryos and D4 embryos demonstrated by immunofluorescent staining. **a** 2-cell embryo from a stressed mouse. **b** 2-cell embryo of a control mouse. **c** D4 embryo from a stressed mouse. **d** D4 embryo of a control mouse. The expression of HSP70 was positive in the embryos from stressed mice, but negative in the control embryos

The IF results showed that HSP70 was distributed mainly in the embryo cytoplasm or around the zona pellucida of the stressed mice (Fig. 1). HSP70 was expressed in 87 % of the 2-cell embryos and 83 % of the D4 embryos of mild stress mice, while for the moderate stress mice these values were 60 and 47 %, respectively; these values were significantly higher than for the control group (*P* < 0.05, Table 3). However, no differences were observed for the 2-cell and D4 embryos of the severe stress group compared with the control group. RT-PCR results similarly showed that compared with the control group, the HSP70 mRNA levels in the 2-cell and D4 embryos of the mild and moderate stress mice were significantly higher, while no differences were observed for the severe stress mice (Table 3).

**Table 3:** Expression of HSP70 in 2-cell and day 4 embryos as measured by immunofluorescence and RT-PCR

	Control group	Mild stress group	Moderate stress group	Severe stress group	*P* value
IF^a^					
Positive 2-cell embryos	10 %(3/30)	87 %(26/30)	60 %(18/30)	13 %(4/30)	<0.05
Positive D4 embryos	20 %(6/20)	83 %(25/30)	47 %(14/30)	17 %(5/30)	<0.05
RT-PCR^b^					
HSP70 of 2-cell embryos	1	6 ± 2*	3 ± 1*	1.2 ± 1	<0.05
HSP70 of D4 embryos	1	13 ± 5*	7 ± 3*	1.5 ± 1	<0.05

^a^Data are shown as rates and were analyzed by *χ* ^2^ test and the partitioning *χ* ^2^ method

^b^Data are shown as the mean ± SD and were analyzed by ANOVA and LSD testing

^*^ *P* < 0.05 compared with the control group, measured by LSD test

### The association between expression of HSP70 in embryos and other variables following CUMS

Statistical analysis showed that the HSP70 mRNA level in the D4 embryos was positively associated with the number of retrieved oocytes, 2-cell embryos, and D4 embryos; the fertilization rate and high quality embryo rate; and the HSP70 mRNA level of the 2-cell embryos (*P* < 0.05, Table 4). We did not find any correlation between the HSP70 mRNA level of the D4 embryos and the blastocyst formation rate, and the body weight of mice.

**Table 4:** Association between expression of HSP70 in embryos and other variables following CUMS

	r	*P* value
No. of oocytes	0.276	<0.05
Fertilization rate	0.492	<0.05
No. of 2-cell embryos	0.325	<0.05
HSP70 mRNA of 2-cell embryos	0.576	<0.05
No. of D4 embryos	0.355	<0.05
No. of high quality embryos	0.423	<0.05
High quality embryo rate	0.537	<0.05
No. of blastocysts	0.413	<0.05
Blastocyst formation rate	0.277	NS
Body weight of mice	0.252	NS

Data were analyzed using linear correlation

## Discussion

In this study, we applied one of the nine stress factors (see ***stress induction***) each day for 4 weeks to develop an effective CUMS female animal model. Under conditions of CUMS, mice showed depressed behavior, similar to the human reaction to chronic and low-intensity adverse events in daily life. Unlike CUMS, chronic restrain stress can’t induce depression-like behaviours but anxiety-like behaviours in mice [29]. The OFT and sucrose consumption test were used to evaluate the validation of the CUMS animal model. The OFT was used to assess locomotor activity and exploratory behavior and the sucrose consumption test was applied to detect anhedonia. According to the data of the time spent in the center of the box, the distance moved, and the rearing count of the OFT, we divided the experimental mice into mild, moderate and severe stress groups for further statistical analysis, which haven’t been reported in other studies yet.

After administration of PMSG/hCG to induce superovulation, we found that moderate or severe stress mice stimulated by CUMS had a decreased ovarian response and oocyte development potential. Tetsushi HIRANO et al. also reported a reproductive suppression male mice model induced by CUMS [30]. This negative outcome resulted from multilevel interactions among the hypothalamic-pituitary- adrenal axis and the hypothalamic-pituitary-gonadal axis, the immune system, and the autonomic nervous system. Elizabeth et al. reported that stress induced increased expression levels of receptors for glucocorticoids, which may cause an increase in gonadotropin inhibitory hormone production, contributing to the hypothalamic suppression of reproductive function [31]. Additionally, chronic unpredictable stress was shown to inhibit the production of intraovarian regulatory factors such as growth and differentiation factor 9 and brain-derived neurotropic factor in follicles [21, 22].

HSC70 expression is known to begin with the onset of zygotic genome activity. It is the predominant HSP70 from the early 2-cell embryo stage to the blastocyst stage. The induction of HSP synthesis by osmotic shock and heat shock begins in 1- or 2-cell embryos and the blastocyst stage, respectively [24, 32]. In our study, we found that the mild stress mice stimulated by CUMS had a similar ovarian response and oocyte development potential compared with the control group. Our IF and RT-PCR showed that HSP70 induced by CUMS in 2-cell and D4 embryos of the mild stress mice were significantly higher than in the control group. Because HSP70 is a stress protective protein, we inferred that the unaffected reproductive function of the mild stress mice might be protected by the overexpression of HSP70. But the mechanism is not clear. Our further study will explore whether HSP70 plays its protective role in embryos according to interact with carboxyl terminal interacting protein (CHIP) [33], and whether cortisol response happens in this condition. On the other hand, in the severe stress group, the ovarian response and oocyte development potential decreased, and the HSP70 mRNA was also low. Severe stress might inhibit proper stress response to generate HSP70 to protect reproduction function, but the correlations with the nervous-endocrine-immune systems are also not clear, which should be studied further.

## Conclusions

HSP70 overexpression may play a protective role in the embryos of mild or moderately stressed mice stimulated by CUMS. But in the severe stress condition, HSP70 level may not be sufficient to exert this protective effect.
